# Ethyl 2-[4-(morpholin-4-yl)phen­yl]-1-[3-(2-oxopyrrolidin-1-yl)prop­yl]-1*H*-1,3-benzimidazole-5-carboxyl­ate monohydrate

**DOI:** 10.1107/S1600536812039268

**Published:** 2012-09-22

**Authors:** Yeong Keng Yoon, Mohamed Ashraf Ali, Tan Soo Choon, Suhana Arshad, Ibrahim Abdul Razak

**Affiliations:** a Institute for Research in Molecular Medicine, Universiti Sains Malaysia, Minden 11800, Penang, Malaysia; b School of Physics, Universiti Sains Malaysia, 11800 USM, Penang, Malaysia

## Abstract

The asymmetric unit of the title compound, C_27_H_32_N_4_O_4_·H_2_O, contains two independent benzimidazole-5-carboxyl­ate mol­ecules and two water mol­ecules. In both main mol­ecules, the pyrrolidine rings are in an envelope conformation with a methyl­ene C atom as the flap. The morpholine rings adopt chair conformations. Both benzimidazole rings are essentially planar, with maximum deviations of 0.008 (1) Å, and form dihedral angles of 37.65 (6) and 45.44 (6)° with the benzene rings. In one mol­ecule, an intra­molecular C—H⋯O hydrogen bond forms an *S*(7) ring motif. In the crystal, O—H⋯O and O—H⋯N hydrogen bonds connect pairs of main mol­ecules and pairs of water mol­ecules into two independent centrosymmetric four-compoment aggregates. These aggregates are connect by C—H⋯O hydrogen bonds leading to the formation of a three-dimensional network, which is stabilized by C—H⋯π interactions.

## Related literature

For the biological activity of benzimidazoles, see: Townsend & Revankar (1970 ▶); Dubey & Sanyal (2010 ▶). For related structures, see: Yoon, Ali, Wei *et al.* (2011 ▶); Yoon *et al.* (2012 ▶); Yoon, Ali, Choon *et al.* (2011 ▶). For ring conformations, see: Cremer & Pople (1975 ▶). For hydrogen-bond motifs, see: Bernstein *et al.* (1995 ▶). For stability of the temperature controller used in the data collection, see: Cosier & Glazer (1986 ▶).
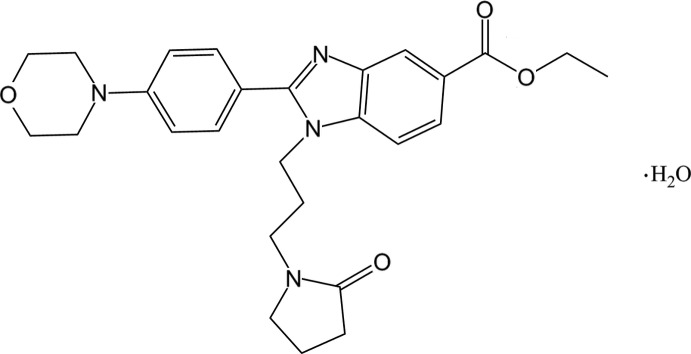


## Experimental

### 

#### Crystal data

C_27_H_32_N_4_O_4_·H_2_O
*M* *_r_* = 494.58
Triclinic, 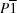

*a* = 12.2602 (3) Å
*b* = 13.7267 (4) Å
*c* = 15.3163 (4) Å
α = 99.357 (1)°
β = 93.700 (1)°
γ = 94.003 (1)°
*V* = 2529.56 (12) Å^3^
*Z* = 4
Mo *K*α radiation
μ = 0.09 mm^−1^
*T* = 100 K
0.67 × 0.40 × 0.11 mm

#### Data collection

Bruker SMART APEXII CCD area-detector diffractometer
Absorption correction: multi-scan (*SADABS*; Bruker, 2009 ▶) *T*_min_ = 0.942, *T*_max_ = 0.991
61724 measured reflections
14611 independent reflections
10995 reflections with *I* > 2σ(*I*)
*R*_int_ = 0.042

#### Refinement

*R*[*F* ^2^ > 2σ(*F* ^2^)] = 0.055
*wR*(*F* ^2^) = 0.152
*S* = 1.03
14611 reflections
667 parameters
H atoms treated by a mixture of independent and constrained refinement
Δρ_max_ = 0.58 e Å^−3^
Δρ_min_ = −0.37 e Å^−3^

### 

Data collection: *APEX2* (Bruker, 2009 ▶); cell refinement: *SAINT* (Bruker, 2009 ▶); data reduction: *SAINT*; program(s) used to solve structure: *SHELXTL* (Sheldrick, 2008 ▶); program(s) used to refine structure: *SHELXTL*; molecular graphics: *SHELXTL*; software used to prepare material for publication: *SHELXTL* and *PLATON* (Spek, 2009 ▶).

## Supplementary Material

Crystal structure: contains datablock(s) global, I. DOI: 10.1107/S1600536812039268/lh5526sup1.cif

Structure factors: contains datablock(s) I. DOI: 10.1107/S1600536812039268/lh5526Isup2.hkl

Supplementary material file. DOI: 10.1107/S1600536812039268/lh5526Isup3.cml

Additional supplementary materials:  crystallographic information; 3D view; checkCIF report

## Figures and Tables

**Table 1:** Hydrogen-bond geometry (Å, °) *Cg*1, *Cg*2, *Cg*3 and *Cg*4 are the centroids of the N1*A*/N2*A*/C1*A*/C6*A*/C7*A*, C21*B*–C26*B*, C21*A*–C26*A* and C21*B*–C26*B* rings, respectively.

*D*—H⋯*A*	*D*—H	H⋯*A*	*D*⋯*A*	*D*—H⋯*A*
C17*A*—H17*B*⋯O3*A*	0.99	2.50	3.2624 (18)	133
O1*WA*—H1*WA*⋯O3*B* ^i^	0.88 (3)	2.00 (3)	2.8652 (18)	168 (2)
O1*WA*—H2*WA*⋯N1*B* ^ii^	0.91 (3)	2.01 (3)	2.9142 (18)	172 (2)
O1*WB*—H1*WB*⋯N1*A*	0.87 (3)	2.05 (3)	2.9142 (19)	172 (2)
O1*WB*—H2*WB*⋯O3*A* ^iii^	0.83 (3)	1.99 (3)	2.8218 (18)	174 (2)
C15*A*—H15*B*⋯O2*B* ^iv^	0.99	2.59	3.412 (2)	141
C15*B*—H15*D*⋯O2*A* ^iv^	0.99	2.50	3.227 (2)	130
C17*A*—H17*A*⋯O3*B* ^i^	0.99	2.44	3.4334 (18)	178
C17*B*—H17*C*⋯O3*A* ^i^	0.99	2.36	3.3274 (18)	166
C25*A*—H25*A*⋯O2*B* ^v^	0.99	2.37	3.309 (2)	159
C26*A*—H26*B*⋯O4*B* ^vi^	0.99	2.54	3.425 (2)	148
C13*B*—H13*B*⋯*Cg*1^iii^	0.95	2.87	3.5419 (15)	129
C21*B*—H21*D*⋯*Cg*2	0.99	2.96	3.8494 (18)	150
C24*A*—H24*B*⋯*Cg*3^iii^	0.99	2.79	3.7712 (19)	172
C24*B*—H24*D*⋯*Cg*4^vii^	0.99	2.67	3.6357 (17)	165

Symmetry codes: (i) 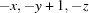; (ii) 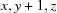; (iii) 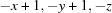; (iv) 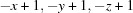; (v) 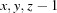; (vi) 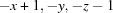; (vii) 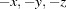.

**Fig. 1.**
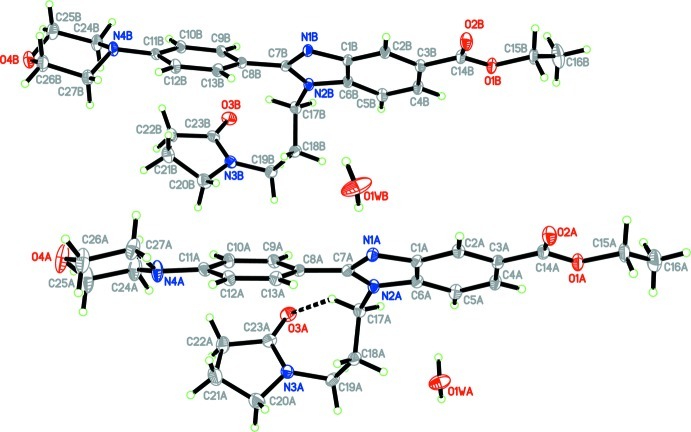
The asymmetric unit of the title compound, showing 50% probability displacement ellipsoids. The dashed line indicates a hydrogen bond.

**Fig. 2.**
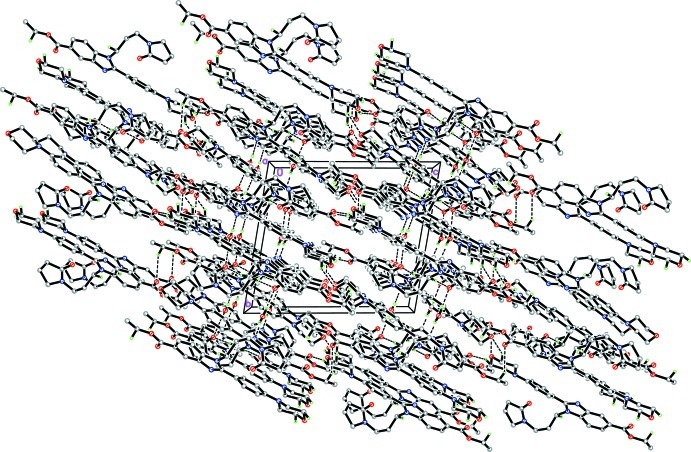
The crystal packing of the title compound. The H atoms not involved in the hydrogen bonds (dashed lines) have been omitted for clarity.

## supplementary crystallographic information

### Comment

Benzimidazoles belong to one of the well known and most extensively studied class of compounds due to their biological activities such as anti-cancer (Townsend & Revankar, 1970) and anthelmintics (Dubey & Sanyal, 2010). As part of our ongoing structural studies of benzimidazole derivatives (Yoon, Ali, Wei *et al.* 2011), we report herein the crystal structure of the title compound.

The asymmetric unit of the title compound, (Fig. 1), consists of two crystallographically independent (*A* and *B*) Ethyl 2-(4-morpholinophenyl)-1-(3-oxopyrolidin-1-yl) propyl)-1*H*-benzo [*d*]imidazole-5-carboxylate and two water molecules. In both molecules, the pyrrolidine rings (N3A/C20A–C23A & N3B/C20B–C23B) are in envelope conformations (Cremer & Pople, 1975) with puckering parameters, Q= 0.2575 (19) Å and φ= 69.4 (4)° in which C21A is the flap and Q= 0.2563 (17) Å and φ= 71.7 (4)° in which C21B is the flap. The morpholine rings, O4A/N4A/C24A–C27A [puckering paramaters; Q = 0.4341 (18) Å, Θ= 1.1 (3)° and φ= 256 (10)°] and O4B/N4B/C24B–C27B [puckering paramaters; Q = 0.5605 (16) Å, Θ= 176.18 (16)° and φ= 153 (3)°] adopt chair conformations. The benzimidazole rings (N1A/N2A/C1A–C7A and N1B/N2B/C1B–C7B) are essentially planar with maximum deviation of 0.008 (1) Å at atom N2A, C6B and C7B. In molecule *A*, the benzimidazole ring, N1A/N2A/C1A–C7A, forms a dihedral of 37.65 (6)° with the benzene (C8A–C13A) ring. The corresponding dihedral angle in molecule *B* is 45.44 (6)°. In molecule *B*, an intramolecular C17A—H17B···O3A hydrogen bond (Table 1) forms *S*(7) ring motif (Bernstein *et al.*, 1995). The bond lengths and angles are within normal ranges and comparable to the related structures (Yoon, Ali, Wei *et al.*, 2011; Yoon *et al.*, 2012; Yoon, Ali, Choon *et al.*, 2011).

In the crystal packing (Fig. 2), intermolecular O—H···O, C—H···N and C—H···O hydrogen bonds (Table 1) link the molecules into a three-dimensional network. The intermolecular C13B—H13B···*Cg*1^iii^, C21B—H21D···*Cg*2, C24A—H24B···*Cg*3^iii^ and C24B—H24D···*Cg*4^vii^ (Table 1) interactions further stabilize the crystal structure (*Cg*1, *Cg*2, *Cg*3 and *Cg*4 are the centroids of N1A/N2A/C1A/C6A/C7A, C21B–C26B, C21A–C26A and C21B–C26B rings, respectively).

### Experimental

Ethyl 3-amino-4-(3(2-oxopyrrolidin-1yl)propylamino)benzoate (0.84 mmol) and sodium metabisulfite adduct of 4-morpholino benzaldehyde (1.68 mmol) were dissolved in DMF. The reaction mixture was reflux at 403K for 2 h. After completion, the reaction mixture was diluted in ethyl acetate (20 ml) and washed with water (20 ml). The organic layer was collected, dried over Na_2_SO_4_ and the evaporated *in vacuo* to yield the product. The product was recrystallized from ethyl acetate. The crystals were then removed and washed twice gently with cold ethyl acetate.

### Refinement

H atoms of the water molecules were located in a difference Fourier map and refined freely [O–H = 0.84 (3)–0.91 (3) Å]. All other H atoms were positioned geometrically [C–H = 0.95 and 0.99 Å] and refined using a riding model with *U*_iso_(H) = 1.2 and 1.5 *U*_eq_(C). A rotating group model was applied to the methyl groups. Two outliner reflections (0 0 1 and -9 - 2 12) were omitted in the final refinement.

### Crystal data

**Table:**

C_27_H_32_N_4_O_4_·H_2_O	*Z* = 4
*M**_r_* = 494.58	*F*(000) = 1056
Triclinic, *P*1	*D*_x_ = 1.299 Mg m^−^^3^
Hall symbol: -P 1	Mo *K*α radiation, λ = 0.71073 Å
*a* = 12.2602 (3) Å	Cell parameters from 9908 reflections
*b* = 13.7267 (4) Å	θ = 2.5–33.5°
*c* = 15.3163 (4) Å	µ = 0.09 mm^−^^1^
α = 99.357 (1)°	*T* = 100 K
β = 93.700 (1)°	Plate, colourless
γ = 94.003 (1)°	0.67 × 0.40 × 0.11 mm
*V* = 2529.56 (12) Å^3^	

### Data collection

**Table:**

Bruker SMART APEXII CCD area-detector diffractometer	14611 independent reflections
Radiation source: fine-focus sealed tube	10995 reflections with *I* > 2σ(*I*)
Graphite monochromator	*R*_int_ = 0.042
φ and ω scans	θ_max_ = 30.0°, θ_min_ = 1.5°
Absorption correction: multi-scan (*SADABS*; Bruker, 2009)	*h* = −17→17
*T*_min_ = 0.942, *T*_max_ = 0.991	*k* = −19→19
61724 measured reflections	*l* = −21→21

### Refinement

**Table:**

Refinement on *F*^2^	Primary atom site location: structure-invariant direct methods
Least-squares matrix: full	Secondary atom site location: difference Fourier map
*R*[*F*^2^ > 2σ(*F*^2^)] = 0.055	Hydrogen site location: inferred from neighbouring sites
*wR*(*F*^2^) = 0.152	H atoms treated by a mixture of independent and constrained refinement
*S* = 1.03	*w* = 1/[σ^2^(*F*_o_^2^) + (0.0734*P*)^2^ + 1.0729*P*] where *P* = (*F*_o_^2^ + 2*F*_c_^2^)/3
14611 reflections	(Δ/σ)_max_ = 0.001
667 parameters	Δρ_max_ = 0.58 e Å^−^^3^
0 restraints	Δρ_min_ = −0.37 e Å^−^^3^

### Special details

**Table:**

Experimental. The crystal was placed in the cold stream of an Oxford Cryosystems Cobra open-flow nitrogen cryostat (Cosier & Glazer, 1986) operating at 100.0 (1) K.
Geometry. All e.s.d.'s (except the e.s.d. in the dihedral angle between two l.s. planes) are estimated using the full covariance matrix. The cell e.s.d.'s are taken into account individually in the estimation of e.s.d.'s in distances, angles and torsion angles; correlations between e.s.d.'s in cell parameters are only used when they are defined by crystal symmetry. An approximate (isotropic) treatment of cell e.s.d.'s is used for estimating e.s.d.'s involving l.s. planes.
Refinement. Refinement of *F*^2^ against ALL reflections. The weighted *R*-factor *wR* and goodness of fit *S* are based on *F*^2^, conventional *R*-factors *R* are based on *F*, with *F* set to zero for negative *F*^2^. The threshold expression of *F*^2^ > σ(*F*^2^) is used only for calculating *R*-factors(gt) *etc*. and is not relevant to the choice of reflections for refinement. *R*-factors based on *F*^2^ are statistically about twice as large as those based on *F*, and *R*- factors based on ALL data will be even larger.

### Fractional atomic coordinates and isotropic or equivalent isotropic displacement parameters (Å^2^)

**Table:**

	*x*	*y*	*z*	*U*_iso_*/*U*_eq_	
O1A	0.66537 (9)	0.88724 (8)	0.48359 (7)	0.0207 (2)	
O2A	0.80726 (9)	0.79642 (8)	0.44866 (7)	0.0229 (2)	
O3A	0.25226 (9)	0.68922 (8)	−0.13551 (8)	0.0235 (2)	
O4A	0.64255 (10)	0.32281 (11)	−0.46338 (9)	0.0396 (4)	
N1A	0.68276 (10)	0.65123 (9)	0.11445 (8)	0.0147 (2)	
N2A	0.52634 (9)	0.71947 (9)	0.08170 (8)	0.0150 (2)	
N3A	0.38761 (10)	0.80132 (9)	−0.16261 (8)	0.0184 (2)	
N4A	0.63208 (11)	0.45033 (11)	−0.29891 (8)	0.0240 (3)	
C1A	0.65487 (11)	0.71291 (10)	0.18925 (9)	0.0138 (3)	
C2A	0.70794 (11)	0.73504 (10)	0.27435 (9)	0.0152 (3)	
H2AA	0.7733	0.7058	0.2887	0.018*	
C3A	0.66221 (11)	0.80115 (10)	0.33738 (9)	0.0157 (3)	
C4A	0.56478 (12)	0.84465 (11)	0.31643 (10)	0.0178 (3)	
H4AA	0.5355	0.8896	0.3611	0.021*	
C5A	0.51084 (12)	0.82345 (11)	0.23243 (10)	0.0182 (3)	
H5AA	0.4455	0.8528	0.2182	0.022*	
C6A	0.55780 (11)	0.75645 (10)	0.16971 (9)	0.0152 (3)	
C7A	0.60465 (11)	0.65698 (10)	0.05185 (9)	0.0142 (3)	
C8A	0.60534 (11)	0.60341 (10)	−0.03932 (9)	0.0141 (3)	
C9A	0.51195 (12)	0.55865 (10)	−0.09139 (9)	0.0165 (3)	
H9AA	0.4421	0.5637	−0.0683	0.020*	
C10A	0.51939 (12)	0.50689 (11)	−0.17616 (9)	0.0172 (3)	
H10A	0.4546	0.4772	−0.2103	0.021*	
C11A	0.62128 (12)	0.49775 (11)	−0.21216 (9)	0.0161 (3)	
C12A	0.71504 (12)	0.54133 (11)	−0.15894 (9)	0.0173 (3)	
H12A	0.7852	0.5356	−0.1814	0.021*	
C13A	0.70707 (11)	0.59237 (10)	−0.07463 (9)	0.0163 (3)	
H13A	0.7719	0.6206	−0.0398	0.020*	
C14A	0.72009 (12)	0.82628 (11)	0.42737 (9)	0.0169 (3)	
C15A	0.71598 (14)	0.91412 (12)	0.57342 (10)	0.0256 (3)	
H15A	0.7853	0.9559	0.5741	0.031*	
H15B	0.7323	0.8540	0.5982	0.031*	
C16A	0.63542 (16)	0.97026 (15)	0.62659 (12)	0.0353 (4)	
H16A	0.6639	0.9859	0.6890	0.053*	
H16B	0.5655	0.9298	0.6218	0.053*	
H16C	0.6240	1.0318	0.6039	0.053*	
C17A	0.43840 (11)	0.75615 (11)	0.02944 (9)	0.0160 (3)	
H17A	0.3789	0.7748	0.0680	0.019*	
H17B	0.4077	0.7024	−0.0188	0.019*	
C18A	0.47845 (12)	0.84531 (11)	−0.01077 (10)	0.0187 (3)	
H18A	0.5468	0.8313	−0.0398	0.022*	
H18B	0.4952	0.9034	0.0369	0.022*	
C19A	0.39221 (13)	0.86937 (11)	−0.07883 (10)	0.0204 (3)	
H19A	0.3194	0.8663	−0.0545	0.025*	
H19B	0.4093	0.9378	−0.0896	0.025*	
C20A	0.45280 (15)	0.82253 (13)	−0.23446 (11)	0.0293 (4)	
H20A	0.5303	0.8422	−0.2127	0.035*	
H20B	0.4232	0.8759	−0.2630	0.035*	
C21A	0.44200 (16)	0.72360 (14)	−0.29895 (12)	0.0328 (4)	
H21A	0.4393	0.7345	−0.3613	0.039*	
H21B	0.5040	0.6835	−0.2880	0.039*	
C22A	0.33378 (14)	0.67318 (13)	−0.27850 (11)	0.0270 (3)	
H22A	0.2727	0.6857	−0.3196	0.032*	
H22B	0.3381	0.6008	−0.2833	0.032*	
C23A	0.31818 (12)	0.71960 (11)	−0.18452 (10)	0.0185 (3)	
C24A	0.53510 (13)	0.41985 (14)	−0.35701 (11)	0.0285 (4)	
H24A	0.4881	0.4759	−0.3547	0.034*	
H24B	0.4935	0.3650	−0.3357	0.034*	
C25A	0.55943 (15)	0.38658 (17)	−0.45059 (11)	0.0380 (5)	
H25A	0.4914	0.3532	−0.4838	0.046*	
H25B	0.5793	0.4460	−0.4770	0.046*	
C26A	0.73703 (15)	0.35354 (15)	−0.40709 (11)	0.0340 (4)	
H26A	0.7760	0.4099	−0.4281	0.041*	
H26B	0.7858	0.2987	−0.4120	0.041*	
C27A	0.71833 (14)	0.38407 (13)	−0.31218 (11)	0.0283 (4)	
H27A	0.6992	0.3242	−0.2862	0.034*	
H27B	0.7872	0.4172	−0.2802	0.034*	
O1B	0.17128 (9)	0.40808 (8)	0.45880 (7)	0.0202 (2)	
O2B	0.32510 (9)	0.33404 (8)	0.42527 (7)	0.0232 (2)	
O3B	−0.23642 (9)	0.17337 (8)	−0.16601 (8)	0.0231 (2)	
O4B	0.12139 (10)	−0.16714 (9)	−0.48934 (7)	0.0254 (2)	
N1B	0.20016 (10)	0.15817 (9)	0.09803 (8)	0.0146 (2)	
N2B	0.04049 (9)	0.21890 (9)	0.06163 (8)	0.0143 (2)	
N3B	−0.09212 (10)	0.28326 (9)	−0.18049 (8)	0.0173 (2)	
N4B	0.13616 (10)	−0.08559 (9)	−0.30449 (8)	0.0177 (2)	
C1B	0.17154 (11)	0.22424 (10)	0.16978 (9)	0.0134 (3)	
C2B	0.22552 (11)	0.25486 (10)	0.25383 (9)	0.0151 (3)	
H2BA	0.2930	0.2298	0.2698	0.018*	
C3B	0.17737 (11)	0.32329 (10)	0.31350 (9)	0.0157 (3)	
C4B	0.07777 (12)	0.36160 (11)	0.28975 (9)	0.0175 (3)	
H4BA	0.0473	0.4086	0.3320	0.021*	
C5B	0.02341 (12)	0.33243 (11)	0.20668 (9)	0.0176 (3)	
H5BA	−0.0434	0.3584	0.1905	0.021*	
C6B	0.07180 (11)	0.26286 (10)	0.14780 (9)	0.0145 (3)	
C7B	0.12087 (11)	0.15719 (10)	0.03483 (9)	0.0138 (3)	
C8B	0.11987 (11)	0.09680 (10)	−0.05387 (9)	0.0141 (3)	
C9B	0.02794 (12)	0.03843 (10)	−0.09653 (9)	0.0156 (3)	
H9BA	−0.0393	0.0395	−0.0690	0.019*	
C10B	0.03271 (12)	−0.02111 (11)	−0.17829 (9)	0.0166 (3)	
H10B	−0.0311	−0.0607	−0.2051	0.020*	
C11B	0.12970 (12)	−0.02411 (10)	−0.22224 (9)	0.0153 (3)	
C12B	0.22229 (12)	0.03535 (11)	−0.17892 (9)	0.0175 (3)	
H12B	0.2894	0.0354	−0.2067	0.021*	
C13B	0.21727 (11)	0.09352 (10)	−0.09678 (9)	0.0163 (3)	
H13B	0.2813	0.1320	−0.0689	0.020*	
C14B	0.23436 (12)	0.35468 (11)	0.40338 (9)	0.0172 (3)	
C15B	0.21555 (13)	0.43897 (12)	0.55009 (10)	0.0223 (3)	
H15C	0.2702	0.4965	0.5547	0.027*	
H15D	0.2516	0.3843	0.5719	0.027*	
C16B	0.12101 (16)	0.46633 (18)	0.60303 (12)	0.0411 (5)	
H16D	0.1471	0.4870	0.6656	0.062*	
H16E	0.0672	0.4090	0.5972	0.062*	
H16F	0.0867	0.5210	0.5811	0.062*	
C17B	−0.05096 (11)	0.24722 (11)	0.00739 (9)	0.0158 (3)	
H17C	−0.1156	0.2538	0.0428	0.019*	
H17D	−0.0706	0.1942	−0.0444	0.019*	
C18B	−0.02321 (13)	0.34433 (11)	−0.02516 (10)	0.0195 (3)	
H18C	0.0522	0.3455	−0.0448	0.023*	
H18D	−0.0259	0.4003	0.0241	0.023*	
C19B	−0.10395 (13)	0.35643 (11)	−0.10218 (10)	0.0206 (3)	
H19C	−0.1798	0.3491	−0.0842	0.025*	
H19D	−0.0910	0.4238	−0.1167	0.025*	
C20B	−0.00713 (13)	0.29695 (12)	−0.24108 (11)	0.0254 (3)	
H20C	0.0648	0.3189	−0.2080	0.030*	
H20D	−0.0262	0.3462	−0.2790	0.030*	
C21B	−0.00526 (13)	0.19367 (13)	−0.29671 (11)	0.0256 (3)	
H21C	0.0088	0.1982	−0.3589	0.031*	
H21D	0.0516	0.1561	−0.2716	0.031*	
C22B	−0.11994 (13)	0.14519 (12)	−0.29112 (10)	0.0217 (3)	
H22C	−0.1693	0.1531	−0.3428	0.026*	
H22D	−0.1175	0.0737	−0.2889	0.026*	
C23B	−0.15789 (11)	0.19944 (11)	−0.20610 (9)	0.0171 (3)	
C24B	0.03344 (13)	−0.13261 (12)	−0.35087 (10)	0.0226 (3)	
H24C	−0.0104	−0.0815	−0.3712	0.027*	
H24D	−0.0095	−0.1652	−0.3095	0.027*	
C25B	0.05476 (14)	−0.20895 (12)	−0.43018 (11)	0.0254 (3)	
H25C	0.0915	−0.2637	−0.4091	0.030*	
H25D	−0.0161	−0.2369	−0.4622	0.030*	
C26B	0.22365 (14)	−0.12744 (12)	−0.44363 (10)	0.0247 (3)	
H26C	0.2709	−0.0998	−0.4852	0.030*	
H26D	0.2613	−0.1810	−0.4212	0.030*	
C27B	0.20697 (14)	−0.04717 (12)	−0.36697 (10)	0.0236 (3)	
H27C	0.2789	−0.0211	−0.3358	0.028*	
H27D	0.1731	0.0081	−0.3898	0.028*	
O1WA	0.34451 (10)	1.00467 (10)	0.12704 (10)	0.0318 (3)	
O1WB	0.83897 (11)	0.50664 (10)	0.14244 (12)	0.0425 (4)	
H1WA	0.303 (2)	0.955 (2)	0.1401 (16)	0.052 (7)*	
H2WA	0.295 (2)	1.048 (2)	0.1147 (17)	0.061 (8)*	
H1WB	0.790 (2)	0.5461 (19)	0.1295 (16)	0.052 (7)*	
H2WB	0.808 (2)	0.450 (2)	0.1384 (17)	0.054 (7)*	

### Atomic displacement parameters (Å^2^)

**Table:**

	*U*^11^	*U*^22^	*U*^33^	*U*^12^	*U*^13^	*U*^23^
O1A	0.0249 (5)	0.0226 (5)	0.0130 (5)	0.0054 (4)	0.0001 (4)	−0.0023 (4)
O2A	0.0218 (5)	0.0278 (6)	0.0175 (5)	0.0051 (4)	−0.0023 (4)	−0.0006 (4)
O3A	0.0185 (5)	0.0212 (5)	0.0304 (6)	−0.0006 (4)	0.0018 (4)	0.0040 (5)
O4A	0.0298 (7)	0.0514 (8)	0.0284 (7)	0.0123 (6)	−0.0044 (5)	−0.0222 (6)
N1A	0.0156 (5)	0.0144 (5)	0.0133 (5)	0.0020 (4)	0.0000 (4)	0.0005 (4)
N2A	0.0155 (5)	0.0162 (5)	0.0127 (5)	0.0034 (4)	−0.0012 (4)	0.0005 (4)
N3A	0.0197 (6)	0.0190 (6)	0.0154 (6)	−0.0020 (5)	−0.0026 (5)	0.0025 (5)
N4A	0.0200 (6)	0.0367 (8)	0.0131 (6)	0.0121 (6)	−0.0037 (5)	−0.0044 (5)
C1A	0.0136 (6)	0.0132 (6)	0.0140 (6)	0.0018 (5)	0.0004 (5)	0.0006 (5)
C2A	0.0134 (6)	0.0169 (6)	0.0151 (7)	0.0018 (5)	−0.0004 (5)	0.0021 (5)
C3A	0.0157 (6)	0.0166 (6)	0.0136 (6)	−0.0002 (5)	0.0000 (5)	0.0004 (5)
C4A	0.0180 (7)	0.0186 (7)	0.0160 (7)	0.0039 (5)	0.0018 (5)	−0.0010 (5)
C5A	0.0154 (6)	0.0200 (7)	0.0184 (7)	0.0056 (5)	−0.0007 (5)	0.0001 (5)
C6A	0.0142 (6)	0.0153 (6)	0.0156 (7)	0.0010 (5)	−0.0004 (5)	0.0018 (5)
C7A	0.0145 (6)	0.0124 (6)	0.0153 (6)	0.0006 (5)	−0.0001 (5)	0.0017 (5)
C8A	0.0166 (6)	0.0124 (6)	0.0131 (6)	0.0019 (5)	0.0000 (5)	0.0020 (5)
C9A	0.0158 (6)	0.0184 (7)	0.0150 (7)	0.0002 (5)	0.0010 (5)	0.0027 (5)
C10A	0.0163 (6)	0.0196 (7)	0.0147 (7)	0.0000 (5)	−0.0019 (5)	0.0011 (5)
C11A	0.0184 (6)	0.0185 (7)	0.0119 (6)	0.0048 (5)	−0.0003 (5)	0.0034 (5)
C12A	0.0152 (6)	0.0213 (7)	0.0159 (7)	0.0040 (5)	0.0018 (5)	0.0035 (5)
C13A	0.0141 (6)	0.0177 (6)	0.0169 (7)	0.0023 (5)	−0.0006 (5)	0.0023 (5)
C14A	0.0184 (6)	0.0167 (6)	0.0146 (7)	0.0002 (5)	0.0016 (5)	0.0005 (5)
C15A	0.0358 (9)	0.0258 (8)	0.0131 (7)	0.0072 (7)	−0.0028 (6)	−0.0033 (6)
C16A	0.0399 (10)	0.0419 (11)	0.0213 (8)	0.0027 (8)	0.0081 (7)	−0.0047 (7)
C17A	0.0135 (6)	0.0189 (7)	0.0152 (7)	0.0038 (5)	−0.0027 (5)	0.0024 (5)
C18A	0.0201 (7)	0.0163 (6)	0.0181 (7)	−0.0003 (5)	−0.0049 (5)	0.0013 (5)
C19A	0.0254 (7)	0.0141 (6)	0.0203 (7)	0.0020 (6)	−0.0060 (6)	0.0012 (5)
C20A	0.0312 (9)	0.0318 (9)	0.0245 (8)	−0.0057 (7)	0.0041 (7)	0.0061 (7)
C21A	0.0381 (10)	0.0381 (10)	0.0214 (8)	0.0013 (8)	0.0082 (7)	0.0010 (7)
C22A	0.0326 (9)	0.0273 (8)	0.0178 (7)	−0.0010 (7)	−0.0050 (6)	−0.0015 (6)
C23A	0.0162 (6)	0.0180 (7)	0.0204 (7)	0.0008 (5)	−0.0052 (5)	0.0036 (5)
C24A	0.0185 (7)	0.0396 (10)	0.0218 (8)	0.0042 (7)	−0.0040 (6)	−0.0094 (7)
C25A	0.0316 (9)	0.0608 (13)	0.0186 (8)	0.0215 (9)	−0.0048 (7)	−0.0074 (8)
C26A	0.0340 (9)	0.0451 (11)	0.0210 (8)	0.0200 (8)	−0.0024 (7)	−0.0054 (7)
C27A	0.0281 (8)	0.0307 (9)	0.0233 (8)	0.0136 (7)	−0.0038 (6)	−0.0065 (7)
O1B	0.0226 (5)	0.0223 (5)	0.0136 (5)	0.0063 (4)	−0.0013 (4)	−0.0044 (4)
O2B	0.0198 (5)	0.0301 (6)	0.0170 (5)	0.0051 (4)	−0.0046 (4)	−0.0032 (4)
O3B	0.0177 (5)	0.0255 (6)	0.0272 (6)	−0.0001 (4)	0.0021 (4)	0.0081 (5)
O4B	0.0312 (6)	0.0290 (6)	0.0141 (5)	0.0029 (5)	0.0024 (4)	−0.0023 (4)
N1B	0.0174 (5)	0.0146 (5)	0.0112 (5)	0.0029 (4)	−0.0010 (4)	−0.0001 (4)
N2B	0.0140 (5)	0.0163 (5)	0.0117 (5)	0.0021 (4)	−0.0019 (4)	−0.0001 (4)
N3B	0.0165 (6)	0.0202 (6)	0.0149 (6)	0.0002 (5)	−0.0010 (4)	0.0037 (5)
N4B	0.0205 (6)	0.0201 (6)	0.0107 (5)	−0.0014 (5)	0.0011 (4)	−0.0017 (4)
C1B	0.0144 (6)	0.0131 (6)	0.0117 (6)	0.0016 (5)	−0.0003 (5)	−0.0003 (5)
C2B	0.0148 (6)	0.0165 (6)	0.0131 (6)	0.0027 (5)	−0.0016 (5)	0.0007 (5)
C3B	0.0158 (6)	0.0174 (6)	0.0128 (6)	0.0018 (5)	−0.0008 (5)	−0.0001 (5)
C4B	0.0167 (6)	0.0199 (7)	0.0147 (7)	0.0051 (5)	0.0008 (5)	−0.0018 (5)
C5B	0.0152 (6)	0.0210 (7)	0.0160 (7)	0.0055 (5)	−0.0004 (5)	0.0003 (5)
C6B	0.0143 (6)	0.0160 (6)	0.0124 (6)	0.0010 (5)	−0.0018 (5)	0.0008 (5)
C7B	0.0153 (6)	0.0122 (6)	0.0133 (6)	0.0001 (5)	−0.0006 (5)	0.0014 (5)
C8B	0.0172 (6)	0.0134 (6)	0.0109 (6)	0.0015 (5)	−0.0011 (5)	0.0009 (5)
C9B	0.0178 (6)	0.0168 (6)	0.0119 (6)	−0.0010 (5)	0.0016 (5)	0.0024 (5)
C10B	0.0184 (6)	0.0172 (6)	0.0128 (6)	−0.0026 (5)	−0.0009 (5)	0.0009 (5)
C11B	0.0206 (7)	0.0153 (6)	0.0096 (6)	0.0016 (5)	−0.0013 (5)	0.0015 (5)
C12B	0.0158 (6)	0.0211 (7)	0.0148 (7)	0.0018 (5)	0.0008 (5)	0.0008 (5)
C13B	0.0153 (6)	0.0170 (6)	0.0155 (7)	0.0002 (5)	−0.0014 (5)	0.0008 (5)
C14B	0.0189 (7)	0.0176 (6)	0.0136 (6)	0.0017 (5)	0.0001 (5)	−0.0017 (5)
C15B	0.0289 (8)	0.0231 (7)	0.0121 (7)	0.0029 (6)	−0.0027 (6)	−0.0045 (5)
C16B	0.0360 (10)	0.0642 (14)	0.0193 (9)	0.0046 (9)	0.0058 (7)	−0.0061 (8)
C17B	0.0132 (6)	0.0197 (7)	0.0142 (6)	0.0026 (5)	−0.0032 (5)	0.0031 (5)
C18B	0.0235 (7)	0.0165 (7)	0.0173 (7)	0.0023 (5)	−0.0059 (6)	0.0014 (5)
C19B	0.0247 (7)	0.0190 (7)	0.0178 (7)	0.0058 (6)	−0.0039 (6)	0.0025 (5)
C20B	0.0234 (8)	0.0283 (8)	0.0242 (8)	−0.0039 (6)	0.0050 (6)	0.0051 (6)
C21B	0.0246 (8)	0.0320 (9)	0.0200 (8)	0.0021 (7)	0.0062 (6)	0.0021 (6)
C22B	0.0247 (7)	0.0249 (8)	0.0147 (7)	0.0007 (6)	−0.0020 (6)	0.0026 (6)
C23B	0.0148 (6)	0.0209 (7)	0.0160 (7)	0.0016 (5)	−0.0042 (5)	0.0062 (5)
C24B	0.0218 (7)	0.0264 (8)	0.0164 (7)	−0.0013 (6)	−0.0006 (6)	−0.0039 (6)
C25B	0.0272 (8)	0.0260 (8)	0.0196 (8)	−0.0015 (6)	0.0000 (6)	−0.0044 (6)
C26B	0.0288 (8)	0.0247 (8)	0.0188 (7)	0.0007 (6)	0.0060 (6)	−0.0023 (6)
C27B	0.0296 (8)	0.0220 (7)	0.0176 (7)	−0.0023 (6)	0.0070 (6)	−0.0012 (6)
O1WA	0.0230 (6)	0.0226 (6)	0.0512 (8)	0.0041 (5)	−0.0005 (6)	0.0104 (6)
O1WB	0.0237 (6)	0.0195 (6)	0.0827 (12)	0.0014 (5)	−0.0133 (7)	0.0104 (7)

### Geometric parameters (Å, º)

**Table:**

O1A—C14A	1.3455 (17)	O2B—C14B	1.2070 (17)
O1A—C15A	1.4515 (18)	O3B—C23B	1.2377 (18)
O2A—C14A	1.2149 (18)	O4B—C25B	1.420 (2)
O3A—C23A	1.2344 (19)	O4B—C26B	1.4235 (19)
O4A—C25A	1.392 (2)	N1B—C7B	1.3247 (17)
O4A—C26A	1.396 (2)	N1B—C1B	1.3869 (17)
N1A—C7A	1.3264 (17)	N2B—C6B	1.3773 (17)
N1A—C1A	1.3864 (17)	N2B—C7B	1.3830 (18)
N2A—C6A	1.3808 (17)	N2B—C17B	1.4642 (17)
N2A—C7A	1.3824 (17)	N3B—C23B	1.3456 (18)
N2A—C17A	1.4669 (17)	N3B—C19B	1.4558 (19)
N3A—C23A	1.3427 (18)	N3B—C20B	1.462 (2)
N3A—C19A	1.4546 (19)	N4B—C11B	1.4080 (17)
N3A—C20A	1.456 (2)	N4B—C24B	1.4591 (18)
N4A—C11A	1.4001 (18)	N4B—C27B	1.468 (2)
N4A—C24A	1.4330 (19)	C1B—C2B	1.3953 (18)
N4A—C27A	1.445 (2)	C1B—C6B	1.4089 (18)
C1A—C2A	1.3959 (18)	C2B—C3B	1.3906 (19)
C1A—C6A	1.4053 (18)	C2B—H2BA	0.9500
C2A—C3A	1.3877 (19)	C3B—C4B	1.4124 (19)
C2A—H2AA	0.9500	C3B—C14B	1.4865 (19)
C3A—C4A	1.4138 (19)	C4B—C5B	1.3824 (19)
C3A—C14A	1.4857 (19)	C4B—H4BA	0.9500
C4A—C5A	1.3852 (19)	C5B—C6B	1.3947 (19)
C4A—H4AA	0.9500	C5B—H5BA	0.9500
C5A—C6A	1.3993 (19)	C7B—C8B	1.4707 (18)
C5A—H5AA	0.9500	C8B—C9B	1.3957 (18)
C7A—C8A	1.4694 (19)	C8B—C13B	1.400 (2)
C8A—C9A	1.3961 (18)	C9B—C10B	1.3867 (19)
C8A—C13A	1.3992 (19)	C9B—H9BA	0.9500
C9A—C10A	1.387 (2)	C10B—C11B	1.404 (2)
C9A—H9AA	0.9500	C10B—H10B	0.9500
C10A—C11A	1.403 (2)	C11B—C12B	1.4115 (19)
C10A—H10A	0.9500	C12B—C13B	1.3835 (19)
C11A—C12A	1.4029 (19)	C12B—H12B	0.9500
C12A—C13A	1.378 (2)	C13B—H13B	0.9500
C12A—H12A	0.9500	C15B—C16B	1.492 (2)
C13A—H13A	0.9500	C15B—H15C	0.9900
C15A—C16A	1.496 (2)	C15B—H15D	0.9900
C15A—H15A	0.9900	C16B—H16D	0.9800
C15A—H15B	0.9900	C16B—H16E	0.9800
C16A—H16A	0.9800	C16B—H16F	0.9800
C16A—H16B	0.9800	C17B—C18B	1.522 (2)
C16A—H16C	0.9800	C17B—H17C	0.9900
C17A—C18A	1.523 (2)	C17B—H17D	0.9900
C17A—H17A	0.9900	C18B—C19B	1.5281 (19)
C17A—H17B	0.9900	C18B—H18C	0.9900
C18A—C19A	1.5271 (19)	C18B—H18D	0.9900
C18A—H18A	0.9900	C19B—H19C	0.9900
C18A—H18B	0.9900	C19B—H19D	0.9900
C19A—H19A	0.9900	C20B—C21B	1.533 (2)
C19A—H19B	0.9900	C20B—H20C	0.9900
C20A—C21A	1.536 (2)	C20B—H20D	0.9900
C20A—H20A	0.9900	C21B—C22B	1.527 (2)
C20A—H20B	0.9900	C21B—H21C	0.9900
C21A—C22A	1.528 (3)	C21B—H21D	0.9900
C21A—H21A	0.9900	C22B—C23B	1.509 (2)
C21A—H21B	0.9900	C22B—H22C	0.9900
C22A—C23A	1.506 (2)	C22B—H22D	0.9900
C22A—H22A	0.9900	C24B—C25B	1.519 (2)
C22A—H22B	0.9900	C24B—H24C	0.9900
C24A—C25A	1.488 (2)	C24B—H24D	0.9900
C24A—H24A	0.9900	C25B—H25C	0.9900
C24A—H24B	0.9900	C25B—H25D	0.9900
C25A—H25A	0.9900	C26B—C27B	1.509 (2)
C25A—H25B	0.9900	C26B—H26C	0.9900
C26A—C27A	1.483 (2)	C26B—H26D	0.9900
C26A—H26A	0.9900	C27B—H27C	0.9900
C26A—H26B	0.9900	C27B—H27D	0.9900
C27A—H27A	0.9900	O1WA—H1WA	0.88 (3)
C27A—H27B	0.9900	O1WA—H2WA	0.91 (3)
O1B—C14B	1.3482 (17)	O1WB—H1WB	0.87 (3)
O1B—C15B	1.4514 (17)	O1WB—H2WB	0.84 (3)
			
C14A—O1A—C15A	115.63 (12)	C25B—O4B—C26B	109.43 (12)
C25A—O4A—C26A	113.58 (13)	C7B—N1B—C1B	105.19 (11)
C7A—N1A—C1A	105.09 (11)	C6B—N2B—C7B	106.63 (11)
C6A—N2A—C7A	106.39 (11)	C6B—N2B—C17B	124.46 (11)
C6A—N2A—C17A	124.43 (11)	C7B—N2B—C17B	128.17 (11)
C7A—N2A—C17A	128.26 (12)	C23B—N3B—C19B	124.77 (13)
C23A—N3A—C19A	124.63 (13)	C23B—N3B—C20B	113.41 (12)
C23A—N3A—C20A	113.69 (13)	C19B—N3B—C20B	121.79 (12)
C19A—N3A—C20A	121.42 (13)	C11B—N4B—C24B	117.25 (12)
C11A—N4A—C24A	118.85 (13)	C11B—N4B—C27B	116.73 (12)
C11A—N4A—C27A	117.46 (12)	C24B—N4B—C27B	110.38 (12)
C24A—N4A—C27A	113.95 (13)	N1B—C1B—C2B	130.21 (12)
N1A—C1A—C2A	129.91 (12)	N1B—C1B—C6B	109.88 (11)
N1A—C1A—C6A	109.98 (12)	C2B—C1B—C6B	119.91 (12)
C2A—C1A—C6A	120.11 (12)	C3B—C2B—C1B	117.82 (12)
C3A—C2A—C1A	117.90 (12)	C3B—C2B—H2BA	121.1
C3A—C2A—H2AA	121.0	C1B—C2B—H2BA	121.1
C1A—C2A—H2AA	121.0	C2B—C3B—C4B	121.27 (12)
C2A—C3A—C4A	121.23 (13)	C2B—C3B—C14B	117.65 (12)
C2A—C3A—C14A	117.83 (12)	C4B—C3B—C14B	121.07 (12)
C4A—C3A—C14A	120.93 (12)	C5B—C4B—C3B	121.69 (13)
C5A—C4A—C3A	121.70 (13)	C5B—C4B—H4BA	119.2
C5A—C4A—H4AA	119.1	C3B—C4B—H4BA	119.2
C3A—C4A—H4AA	119.1	C4B—C5B—C6B	116.50 (13)
C4A—C5A—C6A	116.38 (13)	C4B—C5B—H5BA	121.8
C4A—C5A—H5AA	121.8	C6B—C5B—H5BA	121.8
C6A—C5A—H5AA	121.8	N2B—C6B—C5B	131.51 (13)
N2A—C6A—C5A	131.51 (13)	N2B—C6B—C1B	105.69 (12)
N2A—C6A—C1A	105.81 (12)	C5B—C6B—C1B	122.80 (12)
C5A—C6A—C1A	122.68 (13)	N1B—C7B—N2B	112.61 (12)
N1A—C7A—N2A	112.72 (12)	N1B—C7B—C8B	123.05 (12)
N1A—C7A—C8A	122.28 (12)	N2B—C7B—C8B	124.34 (12)
N2A—C7A—C8A	124.98 (12)	C9B—C8B—C13B	117.60 (12)
C9A—C8A—C13A	117.83 (12)	C9B—C8B—C7B	123.58 (13)
C9A—C8A—C7A	124.42 (13)	C13B—C8B—C7B	118.75 (12)
C13A—C8A—C7A	117.68 (12)	C10B—C9B—C8B	121.37 (13)
C10A—C9A—C8A	121.15 (13)	C10B—C9B—H9BA	119.3
C10A—C9A—H9AA	119.4	C8B—C9B—H9BA	119.3
C8A—C9A—H9AA	119.4	C9B—C10B—C11B	121.43 (13)
C9A—C10A—C11A	120.90 (13)	C9B—C10B—H10B	119.3
C9A—C10A—H10A	119.5	C11B—C10B—H10B	119.3
C11A—C10A—H10A	119.5	C10B—C11B—N4B	121.98 (12)
N4A—C11A—C12A	119.47 (13)	C10B—C11B—C12B	116.94 (12)
N4A—C11A—C10A	122.80 (13)	N4B—C11B—C12B	121.06 (13)
C12A—C11A—C10A	117.70 (13)	C13B—C12B—C11B	121.28 (13)
C13A—C12A—C11A	121.08 (13)	C13B—C12B—H12B	119.4
C13A—C12A—H12A	119.5	C11B—C12B—H12B	119.4
C11A—C12A—H12A	119.5	C12B—C13B—C8B	121.37 (13)
C12A—C13A—C8A	121.31 (13)	C12B—C13B—H13B	119.3
C12A—C13A—H13A	119.3	C8B—C13B—H13B	119.3
C8A—C13A—H13A	119.3	O2B—C14B—O1B	123.61 (13)
O2A—C14A—O1A	123.10 (13)	O2B—C14B—C3B	124.76 (13)
O2A—C14A—C3A	124.59 (13)	O1B—C14B—C3B	111.62 (12)
O1A—C14A—C3A	112.30 (12)	O1B—C15B—C16B	106.61 (13)
O1A—C15A—C16A	106.53 (14)	O1B—C15B—H15C	110.4
O1A—C15A—H15A	110.4	C16B—C15B—H15C	110.4
C16A—C15A—H15A	110.4	O1B—C15B—H15D	110.4
O1A—C15A—H15B	110.4	C16B—C15B—H15D	110.4
C16A—C15A—H15B	110.4	H15C—C15B—H15D	108.6
H15A—C15A—H15B	108.6	C15B—C16B—H16D	109.5
C15A—C16A—H16A	109.5	C15B—C16B—H16E	109.5
C15A—C16A—H16B	109.5	H16D—C16B—H16E	109.5
H16A—C16A—H16B	109.5	C15B—C16B—H16F	109.5
C15A—C16A—H16C	109.5	H16D—C16B—H16F	109.5
H16A—C16A—H16C	109.5	H16E—C16B—H16F	109.5
H16B—C16A—H16C	109.5	N2B—C17B—C18B	112.14 (11)
N2A—C17A—C18A	112.28 (11)	N2B—C17B—H17C	109.2
N2A—C17A—H17A	109.1	C18B—C17B—H17C	109.2
C18A—C17A—H17A	109.1	N2B—C17B—H17D	109.2
N2A—C17A—H17B	109.1	C18B—C17B—H17D	109.2
C18A—C17A—H17B	109.1	H17C—C17B—H17D	107.9
H17A—C17A—H17B	107.9	C17B—C18B—C19B	110.76 (12)
C17A—C18A—C19A	111.11 (12)	C17B—C18B—H18C	109.5
C17A—C18A—H18A	109.4	C19B—C18B—H18C	109.5
C19A—C18A—H18A	109.4	C17B—C18B—H18D	109.5
C17A—C18A—H18B	109.4	C19B—C18B—H18D	109.5
C19A—C18A—H18B	109.4	H18C—C18B—H18D	108.1
H18A—C18A—H18B	108.0	N3B—C19B—C18B	111.28 (12)
N3A—C19A—C18A	112.24 (12)	N3B—C19B—H19C	109.4
N3A—C19A—H19A	109.2	C18B—C19B—H19C	109.4
C18A—C19A—H19A	109.2	N3B—C19B—H19D	109.4
N3A—C19A—H19B	109.2	C18B—C19B—H19D	109.4
C18A—C19A—H19B	109.2	H19C—C19B—H19D	108.0
H19A—C19A—H19B	107.9	N3B—C20B—C21B	103.47 (13)
N3A—C20A—C21A	103.17 (13)	N3B—C20B—H20C	111.1
N3A—C20A—H20A	111.1	C21B—C20B—H20C	111.1
C21A—C20A—H20A	111.1	N3B—C20B—H20D	111.1
N3A—C20A—H20B	111.1	C21B—C20B—H20D	111.1
C21A—C20A—H20B	111.1	H20C—C20B—H20D	109.0
H20A—C20A—H20B	109.1	C22B—C21B—C20B	103.60 (13)
C22A—C21A—C20A	103.56 (14)	C22B—C21B—H21C	111.0
C22A—C21A—H21A	111.0	C20B—C21B—H21C	111.0
C20A—C21A—H21A	111.0	C22B—C21B—H21D	111.0
C22A—C21A—H21B	111.0	C20B—C21B—H21D	111.0
C20A—C21A—H21B	111.0	H21C—C21B—H21D	109.0
H21A—C21A—H21B	109.0	C23B—C22B—C21B	104.39 (12)
C23A—C22A—C21A	104.34 (12)	C23B—C22B—H22C	110.9
C23A—C22A—H22A	110.9	C21B—C22B—H22C	110.9
C21A—C22A—H22A	110.9	C23B—C22B—H22D	110.9
C23A—C22A—H22B	110.9	C21B—C22B—H22D	110.9
C21A—C22A—H22B	110.9	H22C—C22B—H22D	108.9
H22A—C22A—H22B	108.9	O3B—C23B—N3B	124.75 (14)
O3A—C23A—N3A	125.12 (14)	O3B—C23B—C22B	126.85 (13)
O3A—C23A—C22A	126.42 (13)	N3B—C23B—C22B	108.40 (13)
N3A—C23A—C22A	108.45 (13)	N4B—C24B—C25B	111.00 (13)
N4A—C24A—C25A	112.80 (14)	N4B—C24B—H24C	109.4
N4A—C24A—H24A	109.0	C25B—C24B—H24C	109.4
C25A—C24A—H24A	109.0	N4B—C24B—H24D	109.4
N4A—C24A—H24B	109.0	C25B—C24B—H24D	109.4
C25A—C24A—H24B	109.0	H24C—C24B—H24D	108.0
H24A—C24A—H24B	107.8	O4B—C25B—C24B	111.78 (13)
O4A—C25A—C24A	116.21 (15)	O4B—C25B—H25C	109.3
O4A—C25A—H25A	108.2	C24B—C25B—H25C	109.3
C24A—C25A—H25A	108.2	O4B—C25B—H25D	109.3
O4A—C25A—H25B	108.2	C24B—C25B—H25D	109.3
C24A—C25A—H25B	108.2	H25C—C25B—H25D	107.9
H25A—C25A—H25B	107.4	O4B—C26B—C27B	110.79 (13)
O4A—C26A—C27A	115.26 (15)	O4B—C26B—H26C	109.5
O4A—C26A—H26A	108.5	C27B—C26B—H26C	109.5
C27A—C26A—H26A	108.5	O4B—C26B—H26D	109.5
O4A—C26A—H26B	108.5	C27B—C26B—H26D	109.5
C27A—C26A—H26B	108.5	H26C—C26B—H26D	108.1
H26A—C26A—H26B	107.5	N4B—C27B—C26B	110.81 (13)
N4A—C27A—C26A	113.01 (14)	N4B—C27B—H27C	109.5
N4A—C27A—H27A	109.0	C26B—C27B—H27C	109.5
C26A—C27A—H27A	109.0	N4B—C27B—H27D	109.5
N4A—C27A—H27B	109.0	C26B—C27B—H27D	109.5
C26A—C27A—H27B	109.0	H27C—C27B—H27D	108.1
H27A—C27A—H27B	107.8	H1WA—O1WA—H2WA	103 (2)
C14B—O1B—C15B	116.99 (11)	H1WB—O1WB—H2WB	108 (2)
			
C7A—N1A—C1A—C2A	179.95 (15)	C7B—N1B—C1B—C2B	−179.34 (15)
C7A—N1A—C1A—C6A	−0.31 (15)	C7B—N1B—C1B—C6B	0.25 (16)
N1A—C1A—C2A—C3A	179.21 (14)	N1B—C1B—C2B—C3B	179.76 (14)
C6A—C1A—C2A—C3A	−0.5 (2)	C6B—C1B—C2B—C3B	0.2 (2)
C1A—C2A—C3A—C4A	0.2 (2)	C1B—C2B—C3B—C4B	−0.7 (2)
C1A—C2A—C3A—C14A	−178.72 (13)	C1B—C2B—C3B—C14B	179.18 (13)
C2A—C3A—C4A—C5A	−0.1 (2)	C2B—C3B—C4B—C5B	0.5 (2)
C14A—C3A—C4A—C5A	178.81 (14)	C14B—C3B—C4B—C5B	−179.44 (14)
C3A—C4A—C5A—C6A	0.2 (2)	C3B—C4B—C5B—C6B	0.3 (2)
C7A—N2A—C6A—C5A	179.00 (15)	C7B—N2B—C6B—C5B	178.63 (15)
C17A—N2A—C6A—C5A	9.2 (2)	C17B—N2B—C6B—C5B	7.7 (2)
C7A—N2A—C6A—C1A	−0.55 (15)	C7B—N2B—C6B—C1B	−0.42 (15)
C17A—N2A—C6A—C1A	−170.37 (13)	C17B—N2B—C6B—C1B	−171.32 (12)
C4A—C5A—C6A—N2A	179.96 (15)	C4B—C5B—C6B—N2B	−179.76 (15)
C4A—C5A—C6A—C1A	−0.6 (2)	C4B—C5B—C6B—C1B	−0.9 (2)
N1A—C1A—C6A—N2A	0.54 (16)	N1B—C1B—C6B—N2B	0.12 (16)
C2A—C1A—C6A—N2A	−179.68 (13)	C2B—C1B—C6B—N2B	179.75 (13)
N1A—C1A—C6A—C5A	−179.05 (13)	N1B—C1B—C6B—C5B	−179.03 (13)
C2A—C1A—C6A—C5A	0.7 (2)	C2B—C1B—C6B—C5B	0.6 (2)
C1A—N1A—C7A—N2A	−0.05 (16)	C1B—N1B—C7B—N2B	−0.53 (16)
C1A—N1A—C7A—C8A	178.61 (12)	C1B—N1B—C7B—C8B	179.32 (13)
C6A—N2A—C7A—N1A	0.39 (16)	C6B—N2B—C7B—N1B	0.62 (16)
C17A—N2A—C7A—N1A	169.69 (13)	C17B—N2B—C7B—N1B	171.07 (13)
C6A—N2A—C7A—C8A	−178.23 (13)	C6B—N2B—C7B—C8B	−179.23 (13)
C17A—N2A—C7A—C8A	−8.9 (2)	C17B—N2B—C7B—C8B	−8.8 (2)
N1A—C7A—C8A—C9A	141.68 (15)	N1B—C7B—C8B—C9B	133.39 (15)
N2A—C7A—C8A—C9A	−39.8 (2)	N2B—C7B—C8B—C9B	−46.8 (2)
N1A—C7A—C8A—C13A	−35.0 (2)	N1B—C7B—C8B—C13B	−43.5 (2)
N2A—C7A—C8A—C13A	143.50 (14)	N2B—C7B—C8B—C13B	136.29 (14)
C13A—C8A—C9A—C10A	−1.5 (2)	C13B—C8B—C9B—C10B	0.3 (2)
C7A—C8A—C9A—C10A	−178.16 (13)	C7B—C8B—C9B—C10B	−176.69 (13)
C8A—C9A—C10A—C11A	0.1 (2)	C8B—C9B—C10B—C11B	−1.0 (2)
C24A—N4A—C11A—C12A	−170.35 (15)	C9B—C10B—C11B—N4B	178.81 (13)
C27A—N4A—C11A—C12A	45.7 (2)	C9B—C10B—C11B—C12B	0.7 (2)
C24A—N4A—C11A—C10A	7.4 (2)	C24B—N4B—C11B—C10B	11.1 (2)
C27A—N4A—C11A—C10A	−136.52 (16)	C27B—N4B—C11B—C10B	145.42 (14)
C9A—C10A—C11A—N4A	−176.83 (14)	C24B—N4B—C11B—C12B	−170.82 (14)
C9A—C10A—C11A—C12A	1.0 (2)	C27B—N4B—C11B—C12B	−36.54 (19)
N4A—C11A—C12A—C13A	177.14 (14)	C10B—C11B—C12B—C13B	0.2 (2)
C10A—C11A—C12A—C13A	−0.8 (2)	N4B—C11B—C12B—C13B	−177.93 (13)
C11A—C12A—C13A—C8A	−0.6 (2)	C11B—C12B—C13B—C8B	−0.9 (2)
C9A—C8A—C13A—C12A	1.7 (2)	C9B—C8B—C13B—C12B	0.6 (2)
C7A—C8A—C13A—C12A	178.62 (13)	C7B—C8B—C13B—C12B	177.74 (13)
C15A—O1A—C14A—O2A	−1.4 (2)	C15B—O1B—C14B—O2B	−1.9 (2)
C15A—O1A—C14A—C3A	179.14 (13)	C15B—O1B—C14B—C3B	176.96 (12)
C2A—C3A—C14A—O2A	3.4 (2)	C2B—C3B—C14B—O2B	8.8 (2)
C4A—C3A—C14A—O2A	−175.51 (15)	C4B—C3B—C14B—O2B	−171.27 (15)
C2A—C3A—C14A—O1A	−177.09 (12)	C2B—C3B—C14B—O1B	−170.03 (13)
C4A—C3A—C14A—O1A	4.0 (2)	C4B—C3B—C14B—O1B	9.9 (2)
C14A—O1A—C15A—C16A	−172.75 (14)	C14B—O1B—C15B—C16B	−162.12 (15)
C6A—N2A—C17A—C18A	85.14 (17)	C6B—N2B—C17B—C18B	74.78 (17)
C7A—N2A—C17A—C18A	−82.40 (17)	C7B—N2B—C17B—C18B	−94.10 (17)
N2A—C17A—C18A—C19A	168.93 (12)	N2B—C17B—C18B—C19B	163.07 (12)
C23A—N3A—C19A—C18A	94.52 (17)	C23B—N3B—C19B—C18B	100.16 (16)
C20A—N3A—C19A—C18A	−91.63 (17)	C20B—N3B—C19B—C18B	−81.86 (17)
C17A—C18A—C19A—N3A	−76.53 (16)	C17B—C18B—C19B—N3B	−66.92 (17)
C23A—N3A—C20A—C21A	−17.48 (18)	C23B—N3B—C20B—C21B	−16.40 (17)
C19A—N3A—C20A—C21A	168.04 (14)	C19B—N3B—C20B—C21B	165.41 (13)
N3A—C20A—C21A—C22A	24.94 (18)	N3B—C20B—C21B—C22B	24.60 (16)
C20A—C21A—C22A—C23A	−24.16 (18)	C20B—C21B—C22B—C23B	−24.49 (16)
C19A—N3A—C23A—O3A	−3.0 (2)	C19B—N3B—C23B—O3B	−0.6 (2)
C20A—N3A—C23A—O3A	−177.29 (15)	C20B—N3B—C23B—O3B	−178.71 (14)
C19A—N3A—C23A—C22A	176.23 (13)	C19B—N3B—C23B—C22B	178.76 (12)
C20A—N3A—C23A—C22A	1.95 (18)	C20B—N3B—C23B—C22B	0.63 (17)
C21A—C22A—C23A—O3A	−166.17 (15)	C21B—C22B—C23B—O3B	−165.12 (14)
C21A—C22A—C23A—N3A	14.59 (17)	C21B—C22B—C23B—N3B	15.55 (16)
C11A—N4A—C24A—C25A	170.14 (16)	C11B—N4B—C24B—C25B	−170.75 (13)
C27A—N4A—C24A—C25A	−44.7 (2)	C27B—N4B—C24B—C25B	52.27 (17)
C26A—O4A—C25A—C24A	−46.8 (3)	C26B—O4B—C25B—C24B	59.34 (17)
N4A—C24A—C25A—O4A	45.7 (3)	N4B—C24B—C25B—O4B	−55.84 (18)
C25A—O4A—C26A—C27A	47.3 (2)	C25B—O4B—C26B—C27B	−60.60 (17)
C11A—N4A—C27A—C26A	−168.58 (15)	C11B—N4B—C27B—C26B	168.84 (13)
C24A—N4A—C27A—C26A	45.7 (2)	C24B—N4B—C27B—C26B	−53.93 (17)
O4A—C26A—C27A—N4A	−47.1 (2)	O4B—C26B—C27B—N4B	58.54 (18)

### Hydrogen-bond geometry (Å, º)

*Cg*1, *Cg*2, *Cg*3 and *Cg*4 are the centroids of the N1*A*/N2*A*/C1*A*/C6*A*/C7*A*, C21*B*–C26*B*, C21*A*–C26*A* and C21*B*–C26*B* rings, respectively.

**Table:**

*D*—H···*A*	*D*—H	H···*A*	*D*···*A*	*D*—H···*A*
C17*A*—H17*B*···O3*A*	0.99	2.50	3.2624 (18)	133
O1*WA*—H1*WA*···O3*B*^i^	0.88 (3)	2.00 (3)	2.8652 (18)	168 (2)
O1*WA*—H2*WA*···N1*B*^ii^	0.91 (3)	2.01 (3)	2.9142 (18)	172 (2)
O1*WB*—H1*WB*···N1*A*	0.87 (3)	2.05 (3)	2.9142 (19)	172 (2)
O1*WB*—H2*WB*···O3*A*^iii^	0.83 (3)	1.99 (3)	2.8218 (18)	174 (2)
C15*A*—H15*B*···O2*B*^iv^	0.99	2.59	3.412 (2)	141
C15*B*—H15*D*···O2*A*^iv^	0.99	2.50	3.227 (2)	130
C17*A*—H17*A*···O3*B*^i^	0.99	2.44	3.4334 (18)	178
C17*B*—H17*C*···O3*A*^i^	0.99	2.36	3.3274 (18)	166
C25*A*—H25*A*···O2*B*^v^	0.99	2.37	3.309 (2)	159
C26*A*—H26*B*···O4*B*^vi^	0.99	2.54	3.425 (2)	148
C13*B*—H13*B*···*Cg*1^iii^	0.95	2.87	3.5419 (15)	129
C21*B*—H21*D*···*Cg*2	0.99	2.96	3.8494 (18)	150
C24*A*—H24*B*···*Cg*3^iii^	0.99	2.79	3.7712 (19)	172
C24*B*—H24*D*···*Cg*4^vii^	0.99	2.67	3.6357 (17)	165

Symmetry codes: (i) −*x*, −*y*+1, −*z*; (ii) *x*, *y*+1, *z*; (iii) −*x*+1, −*y*+1, −*z*; (iv) −*x*+1, −*y*+1, −*z*+1; (v) *x*, *y*, *z*−1; (vi) −*x*+1, −*y*, −*z*−1; (vii) −*x*, −*y*, −*z*.
